# Effect of Constrained Weight Shift With and Without Trunk Stabilization Training on Balance in Chronic Stroke Patients

**DOI:** 10.7759/cureus.109058

**Published:** 2026-05-17

**Authors:** Janhavi N Kulkarni, Suraj Kanase

**Affiliations:** 1 Department of Neurosciences, Krishna College of Physiotherapy, Krishna Vishwa Vidyapeeth (Deemed to be University), Karad, IND; 2 Department of Neurosciences, Krishna College of Physiotherapy, Krishna Vishwa Vidyapeeth (Deemed to be University), karad, IND

**Keywords:** constrained weight shift, functional mobility training, gait training, neuroplasticity-based learning, postural control, proprioception, sensorimotor integration, stroke rehabilitation, task-oriented training, trunk stabilization

## Abstract

Background

Stroke survivors often exhibit impaired postural control and balance due to trunk weakness and asymmetrical weight-bearing, increasing the risk of falls and limiting functional independence. Targeted rehabilitation strategies are essential to improve static and dynamic balance in chronic stroke patients.

Purpose

This study aimed to compare the effectiveness of constrained weight shift (CWS) training with trunk stabilization versus CWS training alone on static and dynamic balance in chronic stroke patients.

Methods

Thirty chronic stroke patients were randomly assigned to Group A (CWS + trunk stabilization) or Group B (CWS only). Both groups underwent 45-60-minute sessions, five days per week for six weeks, totaling 26 supervised sessions. Training involved weight-shifting exercises, functional tasks, and core strengthening exercises for the experimental group. Balance outcomes were assessed using the Berg Balance Scale (BBS) and Trunk Impairment Scale (TIS) pre- and post-intervention.

Results

Both groups demonstrated improvements in static and dynamic balance; however, Group A showed significantly greater gains in BBS and TIS scores (p < 0.05).

Conclusion

Adding trunk stabilization to CWS training enhances static and dynamic balance recovery in chronic stroke patients.

## Introduction

Stroke is a leading cause of long-term disability worldwide, frequently resulting in hemiparesis, impaired balance, and reduced mobility. Approximately 70-80% of stroke survivors experience balance deficits that limit independence and increase the risk of falls [1]. Following a cerebrovascular accident, many patients develop hemiparesis, which leads to deficits in muscle strength, coordination, and proprioception. Stroke survivors exhibit neuromuscular changes that include weakness, sensory loss, abnormal muscle activation patterns, and altered postural and movement control strategies [2]. Post-stroke, around 35% of chronic stroke patients experience difficulties with standing balance, demonstrate asymmetrical weight distribution, impaired weight-shifting ability, or gait abnormalities [3]. Patients may also experience falls, difficulty performing activities of daily living, or require external assistance for such tasks [4]. Therefore, one of the main goals of stroke rehabilitation is to improve balance and gait and to lower the risk of falls in patients [5]. These impairments contribute to asymmetrical postural alignment, characterized by decreased weight-bearing through the paretic lower limb and compensatory overloading of the non-paretic side [6]. Such asymmetry in standing and movement not only reduces the efficiency of gait but also increases energy expenditure and the risk of falls. Research suggests that up to 80% of stroke survivors exhibit persistent balance dysfunction that compromises independence in daily living activities [7].

Since weight-bearing is an essential element of basic mobility activities such as sit-to-stand, standing balance, and walking, one of the most critical goals of physical therapy is to improve weight-bearing in stroke patients [8]. A rapid increase in symmetrical weight-bearing is essential for independence and social participation. Various therapeutic approaches have been used to help stroke patients overcome asymmetrical weight-bearing. The ability to maintain and control balance depends on the integration of sensory input, motor output, and cognitive processes. In stroke survivors, impaired weight shifting toward the paretic limb has been attributed to a combination of abnormal muscle tone, reduced proprioceptive feedback, poor anticipatory postural adjustments, and trunk instability [9].

The resulting asymmetrical stance leads to lateral displacement of the center of mass toward the non-paretic side, reducing the functional contribution of the affected limb. Therefore, therapeutic interventions that facilitate symmetrical weight-bearing and enhance the patient’s ability to shift weight toward the paretic limb are fundamental in neurorehabilitation. Constrained weight shift (CWS) training is one such approach that seeks to improve weight distribution through biomechanical modification or feedback-based strategies.

CWS interventions can be implemented by introducing a heel lift or wedge insert under the paretic or non-paretic limb to encourage active loading of the affected side. The use of a heel lift (2-5 cm) under the paretic limb alters the alignment of the lower limb joints, facilitating lateral weight transfer and increasing ground reaction forces on the affected side [10]. The altered sensory input from the foot and ankle stimulates proprioceptive pathways, potentially enhancing postural awareness and balance control. This mechanical adjustment also helps to address compensatory movement strategies often seen after stroke, such as weight avoidance and trunk lean toward the unaffected side. Studies have demonstrated that constrained weight-bearing tasks can lead to improvements in symmetry indices, standing balance, and gait performance [11].

However, lower limb loading alone may not be sufficient to ensure postural stability if the trunk remains unstable. The trunk acts as a central unit for maintaining balance and coordinating limb movements. Impairment in trunk control following stroke is associated with delayed activation of core muscles, reduced anticipatory postural adjustments, and abnormal trunk sway [12]. Trunk stabilization training (TST) is designed to improve core muscle strength, intersegmental control, and alignment, which are critical for maintaining both static and dynamic balance. Several studies have confirmed that improved trunk performance correlates with better sitting and standing balance, enhanced walking ability, and greater independence in activities of daily living [13].

Despite the individual benefits of CWS and TST, their combined effects have not been adequately explored in chronic stroke rehabilitation. Integrating CWS with TST may produce synergistic effects. While CWS promotes active engagement of the paretic limb and recalibration of postural symmetry, TST enhances proximal control necessary for dynamic stability. Understanding this interaction can provide valuable insights into comprehensive static and dynamic balance retraining strategies.

Thus, the present study aims to investigate the effect of constrained weight shift with and without TST on static and dynamic balance in chronic stroke patients.

## Materials and methods

This was a pre-post experimental comparative study conducted in the Neuroscience Intensive Care Unit, Krishna College of Physiotherapy, over a period of six months during the year 2025. The Institutional Ethics Committee approved the study. Patients with chronic stroke fulfilling the inclusion criteria were briefed about the purpose and nature of the study, and written informed consent was obtained before enrollment.

Participants were then equally assigned to two groups in a 1:1 ratio. The study included individuals diagnosed with chronic stroke (≥6 months post-onset), aged between 40 and 70 years, including both males and females, with unilateral hemiparesis due to ischemic or hemorrhagic stroke. Participants were required to stand independently for at least one minute with or without minimal support and demonstrate a Brunnstrom recovery stage of III or higher in the lower limb, indicating partial voluntary motor control. Participants were excluded if they had severe lower limb spasticity (Modified Ashworth Scale score >3) [14], severe cognitive deficits, neglect, or receptive aphasia affecting comprehension, or orthopedic complications such as fractures, joint deformities, or contractures of the lower limbs.

Participants were divided into an experimental group receiving constrained weight shift training with trunk stabilization and a control group receiving constrained weight shift training alone. The intervention lasted six weeks, with five sessions per week of 45-60 minutes each, totaling 30 supervised sessions conducted by a physiotherapist, with assessments carried out at baseline and post-intervention.

In the experimental group, constrained weight shift training involved placing a 2-5 cm heel lift or wedge under the paretic limb to promote active weight-bearing. Training progressed from static postural tasks with visual feedback to dynamic weight-shifting and functional activities, including sit-to-stand, mini squats, and walking. Trunk stabilization exercises targeted core strength and coordination through bridging, abdominal hollowing, quadruped holds, dynamic trunk movements, and advanced balance activities on unstable surfaces with gradual progression. Conventional training, provided to both groups, included range-of-motion exercises, stretching, lower limb strengthening, balance and gait training, and functional mobility tasks aimed at improving postural control and overall mobility.

The first primary variable for measuring the effect of the intervention was the Berg Balance Scale (BBS). It is an instrument used to assess functional balance and posture and comprises 14 items, with a total score of 56, with each item rated from 0 to 4. It has high reliability (intrarater and interrater ICC = 0.98) [15]. The second primary variable was the Trunk Impairment Scale (TIS). It is an instrument used to evaluate static and dynamic sitting balance and trunk coordination, comprising 23 items, with high reliability (interrater ICC = 0.96-0.97; intrarater ICC = 0.97-0.99) [16].

Post-intervention assessment was conducted, after which data for all variables were collected and entered into an MS Office Excel sheet (Microsoft Corporation, Redmond, Washington). Mean values and standard deviations were calculated for statistical analysis. Data analysis was performed using GraphPad InStat (trial version 3.0632). Normality of data was assessed using the Kolmogorov-Smirnov test, and paired-sample t-tests were used to compare pre- and post-intervention values. A p-value of <0.05 was considered statistically significant.

## Results

**Figure 1:**
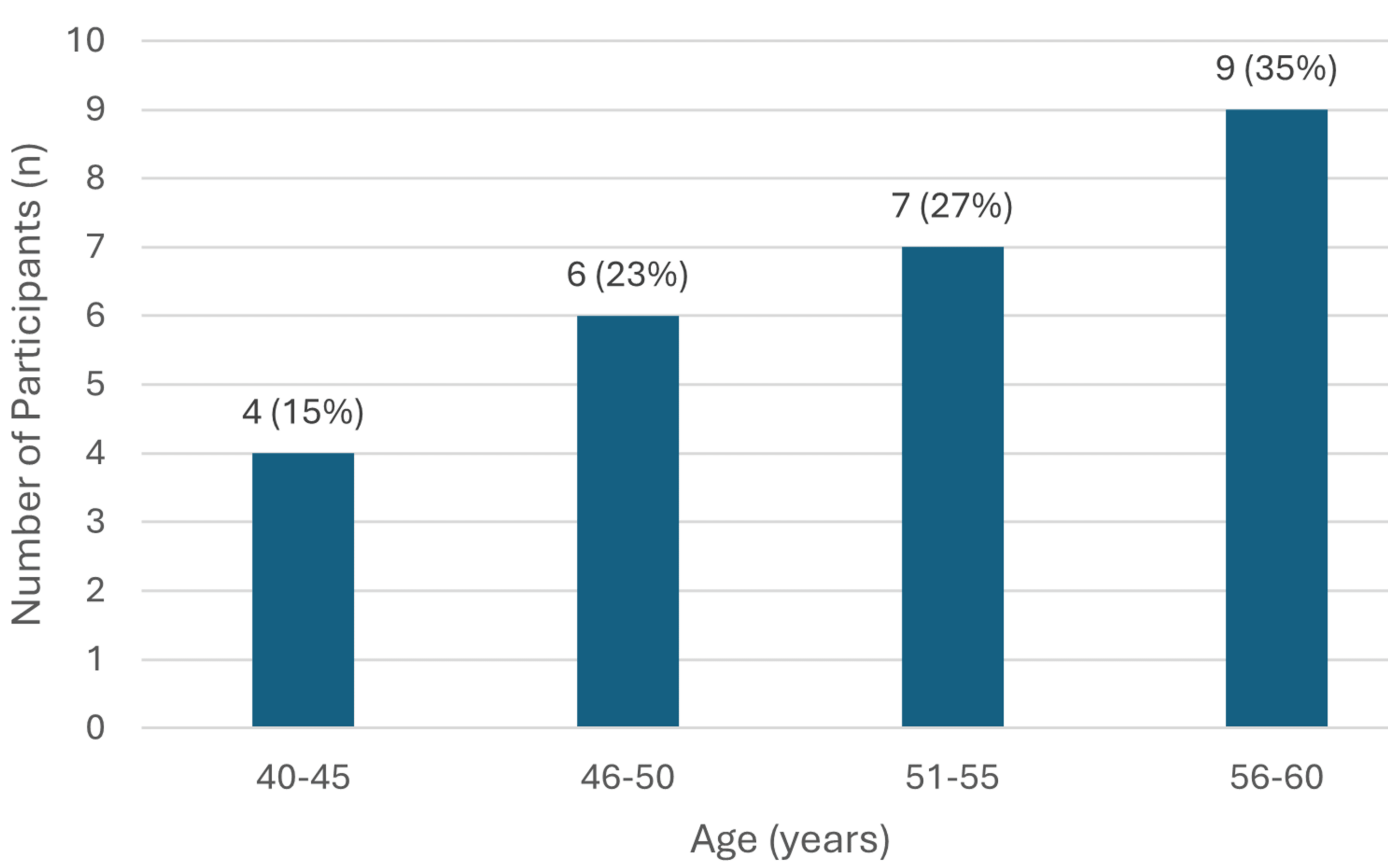
Age-wise distribution of subjects between both groups The proportion of subjects in the age groups of 40-45 years was 15%, 46-50 years was 23%, 51-55 years was 27%, and 56-60 years was 35%.

A total number of 35 male and 20 female patients of age between 40 and 60 years who were suffering from either hemorrhagic or ischemic stroke volunteered for the study; among those, 20 men and 6 women have undergone four weeks of programmed. The age-wise distribution of participants is shown in Figure 1.

Table 1 shows the within-group analyses of BBS and TIS for Groups A and B. In which, Group A showed marked improvement in both BBS and TIS scores with extremely significant results (p < 0.0001), indicating the strong effectiveness of the intervention. Group B demonstrated moderate improvement, with statistically significant changes in BBS (p = 0.021) and TIS (p = 0.0086). 

**Table 1:** Within-group comparison of pre- and post-intervention scores for BBS and TIS in Group A and Group B BBS: Berg Balance Scale; TIS: Trunk Impairment Scale.

Outcome Measure	Group	Phase	Mean	SD	t-value	p-value	Result	Mean Difference
BBS	Group A	Pre	13.4	5.1	8.819	<0.0001	Extremely significant	16.6
		Post	48.1	4.5				
	Group B	Pre	29	4.8	2.45	0.021	Considered significant	4.7
		Post	33.7	5.0				
TIS	Group A	Pre	8.4	2.2	8.66	<0.0001	Extremely significant	10.1
		Post	18.6	3.5				
	Group B	Pre	8.9	1.6	2.86	0.0086	Very significant	1.7
		Post	10.6	1.5				

Table 2 shows between group analysis for Groups A and B. In which, Group A demonstrated substantially greater improvement than Group B in both BBS and TIS scores, with extremely significant differences (p < 0.0001). The mean improvement in Group A was much higher compared to Group B. 

**Table 2:** Between-group comparison of mean differences in BBS and TIS scores between Group A and Group B BBS: Berg Balance Scale; TIS: Trunk Impairment Scale.

Outcome Measure	Group	N	Mean	SD	Mean Difference	t-value	p-value	Result
BBS	Group A	13	16.6	3.4	-11.9	10.45	<0.0001	Extremely significant
	Group B	13	4.7	2.2				
TIS	Group A	13	10.1	3.7	-8.38	7.939	<0.0001	Extremely significant
	Group B	13	1.7	0.7				

## Discussion

The present study investigated the effects of CWS training with and without TST on static and dynamic balance in chronic stroke patients. The findings demonstrated that both groups showed improvement following intervention; however, participants receiving combined CWS and TST exhibited significantly greater improvements in BBS and TIS scores compared to those receiving CWS alone. These results suggest that integrating trunk stabilization exercises with weight-shift training enhances postural control and balance recovery in chronic stroke rehabilitation.

Chronic stroke survivors often present with asymmetric weight distribution, favoring the non-paretic limb due to weakness, sensory deficits, and impaired motor control on the affected side [17]. This asymmetry not only compromises postural stability but also contributes to inefficient gait patterns, increased energy expenditure, and a higher risk of falls. CWS, achieved through the application of a heel lift or wedge insert under the paretic limb, provides a mechanical cue that encourages loading of the affected limb. This intervention increases sensory input from plantar receptors, enhances proprioceptive feedback, and promotes active engagement of the paretic limb [18]. Consequently, patients develop more symmetrical postural strategies and improved center of mass control.

Trunk stabilization plays a crucial role in postural control, particularly in stroke patients who often exhibit core weakness and impaired anticipatory postural adjustments [16]. Incorporating exercises such as bridging, pelvic tilting, trunk rotation, and reaching tasks strengthens deep trunk muscles, enhances intersegmental coordination, and stabilizes the proximal body segments. This proximal control allows more efficient distal movement of the limbs, facilitating improved weight shift and functional balance during both static and dynamic activities [16]. The combination of CWS with TST likely promotes sensorimotor integration by linking improved proprioceptive input from the lower limb with enhanced trunk control, which supports anticipatory and reactive postural adjustments.

Improvements observed in static balance are consistent with findings demonstrating enhanced postural symmetry in hemiparetic patients following weight-bearing training on the paretic limb [19]. Similarly, improvements in dynamic balance, as evidenced by enhanced scores on functional measures such as the BBS, are consistent with previous research demonstrating that trunk-focused exercises combined with weight-bearing activities significantly enhance gait stability, step symmetry, and overall functional mobility. The present findings also indicate that combining CWS training with trunk stabilization may positively influence proprioceptive acuity, as individuals are required to integrate sensory input from both the lower limbs and trunk simultaneously during postural control and functional task performance [20].

Clinically, these findings underscore the importance of integrating trunk stability into rehabilitation programs that use mechanical cues such as heel lifts or wedges. Simple, low-cost interventions such as a 2-5 cm heel lift can be combined effectively with targeted core exercises to produce meaningful functional improvements. These strategies can be implemented in outpatient, inpatient, or home-based rehabilitation settings, allowing therapists to individualize training intensity and complexity based on patient tolerance.

However, the present study has certain limitations. The sample size was relatively small, which may limit the generalizability of the findings. Additionally, the intervention duration was limited to six weeks, and long-term follow-up was not conducted to determine the retention of functional gains. Future studies with larger sample sizes, longer follow-up periods, and objective assessment tools such as electromyography or motion analysis are recommended to further evaluate the effectiveness of combined CWS and TST interventions.

## Conclusions

The study demonstrates that CWS combined with TST significantly improves balance, trunk stability, and postural symmetry in individuals with chronic stroke compared to CWS training alone. These findings indicate that integrating trunk-focused interventions enhances overall postural control and promotes more symmetrical weight-bearing during functional activities.

Furthermore, this combined approach has the potential to improve functional independence and reduce the risk of falls in stroke patients. Therefore, incorporating CWS along with trunk stabilization exercises into routine stroke rehabilitation programs may lead to better clinical outcomes and more effective recovery.
